# Duplication of the dystroglycan gene in most branches of teleost fish

**DOI:** 10.1186/1471-2199-8-34

**Published:** 2007-05-17

**Authors:** Ernesto Pavoni, Davide Cacchiarelli, Roberta Tittarelli, Massimiliano Orsini, Antonio Galtieri, Bruno Giardina, Andrea Brancaccio

**Affiliations:** 1 CNR, Istituto di Chimica del Riconoscimento Molecolare c/o Istituto di Biochimica e Biochimica Clinica, Università Cattolica del Sacro Cuore, Largo F. Vito 1, 00168 Roma, Italy; 2 CRS4 Bioinformatic Unit Parco Scientifico e Tecnologico POLARIS 09010 Pula (CA), Italy; 3 Dipartimento di Chimica Organica e Biologica, Università di Messina, 98122 Messina, Italy

## Abstract

**Background:**

The dystroglycan (DG) complex is a major non-integrin cell adhesion system whose multiple biological roles involve, among others, skeletal muscle stability, embryonic development and synapse maturation. DG is composed of two subunits: α-DG, extracellular and highly glycosylated, and the transmembrane β-DG, linking the cytoskeleton to the surrounding basement membrane in a wide variety of tissues. A single copy of the DG gene (*DAG1*) has been identified so far in humans and other mammals, encoding for a precursor protein which is post-translationally cleaved to liberate the two DG subunits. Similarly, *D. rerio *(zebrafish) seems to have a single copy of *DAG1*, whose removal was shown to cause a severe dystrophic phenotype in adult animals, although it is known that during evolution, due to a whole genome duplication (WGD) event, many teleost fish acquired multiple copies of several genes (paralogues).

**Results:**

Data mining of pufferfish (*T. nigroviridis *and *T. rubripes*) and other teleost fish (*O. latipes *and *G. aculeatus*) available nucleotide sequences revealed the presence of two functional paralogous DG sequences. RT-PCR analysis proved that both the DG sequences are transcribed in *T. nigroviridis*. One of the two DG sequences harbours an additional mini-intronic sequence, 137 bp long, interrupting the uncomplicated exon-intron-exon pattern displayed by *DAG1 *in mammals and *D. rerio*. A similar scenario emerged also in *D. labrax *(sea bass), from whose genome we have cloned and sequenced a new DG sequence that also harbours a shorter additional intronic sequence of 116 bp. Western blot analysis confirmed the presence of DG protein products in all the species analysed including two teleost Antarctic species (*T. bernacchii *and *C. hamatus*).

**Conclusion:**

Our evolutionary analysis has shown that the whole-genome duplication event in the Class Actinopterygii (ray-finned fish) involved also *DAG1*. We unravelled new important molecular genetic details about fish orthologous DGs, which might help to increase the current knowledge on DG expression, maturation and targeting and on its physiopathological role in higher organisms.

## Background

Dystroglycan (DG) is a cell surface adhesion complex, originally isolated from rabbit skeletal muscle, representing the pivotal element of a multimeric complex defined as dystrophin-glycoprotein complex (DGC). In mammals *DAG1 *possesses an uncomplicated exon-intron-exon structure, and its transcription and translation generates a precursor protein that is post-translationally cleaved into two noncovalently associated subunits: the highly glycosylated extracellular α-DG and the transmembrane β-DG [1]. The DG subunits are believed to establish a molecular bridge linking the extracellular matrix to the cytoskeleton [2]. In skeletal muscle and in a wide variety of tissues α-DG binds extracellular matrix molecules, such as laminins, agrins and perlecan, and interacts non covalently with β-DG, that binds dystrophin via its cytoplasmic tail [3]. Several cDNA sequences, which in most cases correspond to a highly conserved protein product 895 aa long, have been reported in different organisms such as human, mouse, dog, amphibia and fish DGs. The degree of sequence identity among mammals is remarkably high (> 90%), while the recently identified cDNA sequences of *X. laevis *and *D. rerio *(zebrafish) confirm that a very high degree of similarity is found also in lower vertebrate species [4,5].

DG is believed to have an increasingly important role in human health, being involved in pathological processes ranging from cancer progression to infective diseases [6]. In particular, in human skeletal muscle DG, as well as several proteins belonging to the DGC (like dystrophin and sarcoglycans), is involved in severe forms of muscular diseases [7]. On the other hand, until now there are no reports about muscular diseases directly generated by *DAG1 *mutations (primary dystroglycanopathies), not surprisingly since the DG knockout experiment in mice causes an early arrest of the embryonic development (at day 6.5), due to the disruption of the Reichert's membrane [8]. However, in particular muscular diseases, known as congenital dystrophies (Muscle-Eye-Brain disease, MEB; Fukuyama Congenital Muscular Dystrophy, FCMD; Walker-Warburg Syndrome, WWS), mutations in different genes encoding for glycosyltransferases are regarded to generate an abnormal glycosylation of α-DG [9-11]. This alteration of the glycosylation pattern of α-DG compromises its binding to extracellular matrix molecules and it is thought to be the reason for the progressive muscle fibre degeneration; this kind of human congenital disorders have been defined as "secondary dystroglycanopathies" [12].

In the last years, a large body of knowledge originated from comparative biochemical and physiological studies about dystroglycan and the dystrophin glycoprotein complex in *D. rerio *[5,13-15], which showed that DG indeed plays a crucial role for adult skeletal muscle stability [5]. With the aim of carrying out an expanded genetic and biochemical comparative analysis, we examined *DAG1 *from several fish species; besides *D. labrax *(sea bass) and *D. rerio *(zebrafish), we also analysed pufferfish characterized by compact genomes (*T. nigroviridis *and *T. rubripes*), other teleosts such as *O. latipes *(medaka) and *G. aculeatus *(stickleback), and Antarctic species (*T. bernacchii *and *C. hamatus*). So far it was generically assumed that all vertebrate species would share only one copy of *DAG1*, even if a whole genome duplication (WGD) event, involving a large number of genes, has been described in Actinopterygii [16].

Although a DG gene duplication event has not been identified in *D. rerio *[5], our computer mining of genomic data available for pufferfish indicates that two different DG sequences are present. Accordingly, via the analysis of DNAs (and cDNAs) from *T. nigroviridis*, we identified two functional paralogous DG sequences, hereinafter defined as *DAG1a *and *DAG1b*. Moreover, for the first time we have cloned and sequenced *DAG1 *in sea bass (*D. labrax*), showing that it contains an additional mini-intronic sequence of about 150 bp, which is properly spliced out upon transcription.

## Results

### Analysis of DAG1a and DAG1b sequences

**Figure 1:**
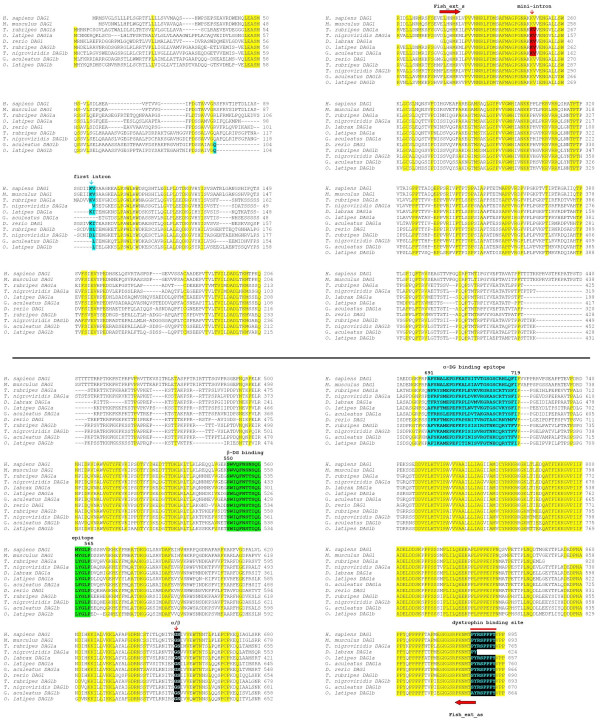
Multiple alignment of dystroglycan amino acid sequences obtained using the ClustalW software. The DG protein sequences from *T. rubripes*, *T. nigroviridis*, *O. latipes *and *G. aculeatus *are the conceptual translations of genomic available DNA sequences. Identical residues are highlighted in yellow. The cyan highlighting identifies the first intron insertion site and the red highlighting identifies the insertion site of the mini-intron. It should be noted that due to some possible sequencing mistakes, the 3' end of *T. rubripes DAG1a*, and therfore the corresponding C-terminal amino acid sequence, is not fully available in the Ensembl databank. The α/β cleavage site is also highlighted (black) while the green highlighting identifies the β-DG binding epitope and the cyan one the α-DG binding epitope [39,40]. The regions chosen for designing the two primers (FISH_ext_s and FISH_ext_as) used for the DG-homologous cloning experiment in *D. labrax *are indicated by red arrows.

Two paralogous genomic DNA sequences, for *T. rubripes *and *T. nigroviridis *respectively, were found at the Ensembl database  already catalogued as *DAG1 *under the GenBank accession numbers reported in Table 1. In the same databank other DG sequences were also found for *O. latipes *and *G. aculeatus*. Using the program ClustalW, we have obtained a multiple alignment of the newly identified fish DG sequences together with those from human and mouse (Fig. 1). In addition, we have reported (Table 2) the reciprocal scores originating from the ClustalW alignment in Fig. 1, from which it can be seen that the similarity is higher between the orthologues of DAG1a or DAG1b proteins that between paralogous DG proteins within the same species (Table 2).

The *T. rubripes DAG1a *is 5674 bp long, starting from the putative transcription start site up to the stop codon, and consists of three exons separated by two introns. The same structure is present in *T. nigrovoridis DAG1a*, although the 5' region has not yet been clearly annotated in the Ensembl database. The 5' region of *G. aculeatus DAG1a *gene is still partially defined as well, even if we have been able to recognize the typical "exon2-mini-intron-exon3" structure. This arrangement is also present in *O. latipes DAG1a*, although a peculiar feature of this gene is its very long second intron spanning ≈ 22 kb (Table 1). *DAG1b *paralogous genes from pufferfish (*T. nigrovoridis *and *T. rubripes*), from *G. aculeatus *and *O. latipes *contain two exons, of 310–360 and ~ 2300 bp respectively, separated by only one intron (800–1000 bp) which is much shorter than the one typically present in human (19977 bp) or zebrafish (14512 bp) gene sequences (see Table 1).

### Cloning of the D. labrax DG gene

The gene fishing experiment that allowed the cloning of the *D. labrax *(sea bass) *DAG1 *was performed using a primer pair, FISH_ext_s and FISH_ext_as (Table 3), specifically designed in order to closely match two highly conserved regions identified by aligning the *DAG1 *sequences from *D. rerio *and *T. rubripes *(see Fig. 1). The PCR reaction, performed using genomic DNA extracted from *D. labrax *skeletal muscle as template, produced a fragment of ~ 2000 bp, displaying a longer size than the one expected from the *D. rerio *and *T. rubripes *DG sequences. Indeeed, cloning and sequencing of this fragment revealed the presence of an additional 116 bp mini-intron. The nucleotides at the splice site of the exon/mini-intron boundary conformed to the GT-AG rule. The newly identified DG sequence from *D. labrax *is 1990 bp long; it encodes for a 624 amino acids sequence (deposited under the accession number DQ149510), spanning a portion of the α-DG N-terminal region and protruding through almost the entire β-DG region. The amino acid alignment between this sequence and that of DG from *D. rerio *shows a 76.9% identity in a 631 residues overlap (Fig. 1). Based on the alignment score with other vertebrate sequences, our new DG sequence from *D. labrax *is likely to correspond to the *DAG1a *family of sequences (see Table 2).

### Paralogous pufferfish DAG genes are correctly spliced

**Figure 2:**
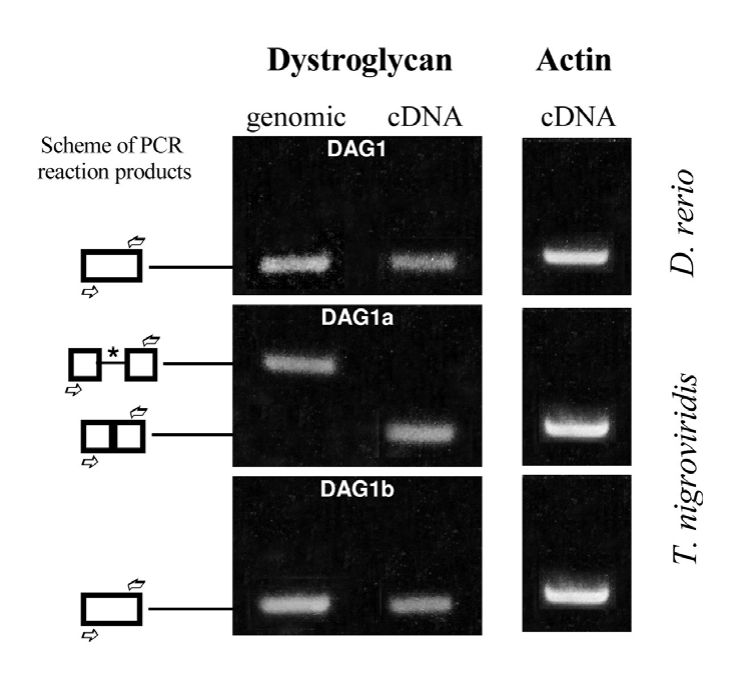
1% agarose gel electrophoresis of the PCR reaction products, carried out using a couple of specific primers (whose sequences are reported in Table 1) flanking the mini-intron region of *DAG1a *and its counterpart region in *DAG1b *from *T. nigroviridis*. The cartoon on the left refers to the different reaction products obtained with appropriate primers (depicted as arrows). The mini-intron is evidenced by an asterisks. The two DNA fragments obtained using respectively genomic or cDNA as a template, differ for the presence of the mini-intron sequence, demonstrating the splicing-out of the mini-intron sequence in *T. nigroviridis*.

PCR analysis suggested that the mini-intron sequence of 137 bp would be properly spliced in *T. nigroviridis*, since shorter bands emerged from the analysis of cDNA samples with respect of genomic DNAs (Fig. 2). This hypothesis was strongly supported by the evidence of a conservation of several typical intron consensus sequences, such as the donor, the acceptor splice site, the branching site and the typical pyrimidin rich-region too (data not shown). A PCR experiment carried out using a primer pair flanking the mini-intron region (see Table 3), produced two fragments displaying a different size when genomic DNA or retro-transcribed cDNA were respectively used as template. The two DNA fragments differ for the presence of the mini-intron sequence, confirming the splicing-out of the mini-intron sequence within the RNA. RT-PCR demonstrated that both *DAG1a *and *DAG1b *genes are transcribed and are likely to be expressed. In *D. rerio *the amplified fragment shows the expected size confirming the presence of a single *DAG1 *in zebrafish and the actin control experiment demonstrated that the cDNA was totally free from any possible genomic DNA contamination (Fig. 2).

### Western blot analysis of β-DG

**Figure 3:**
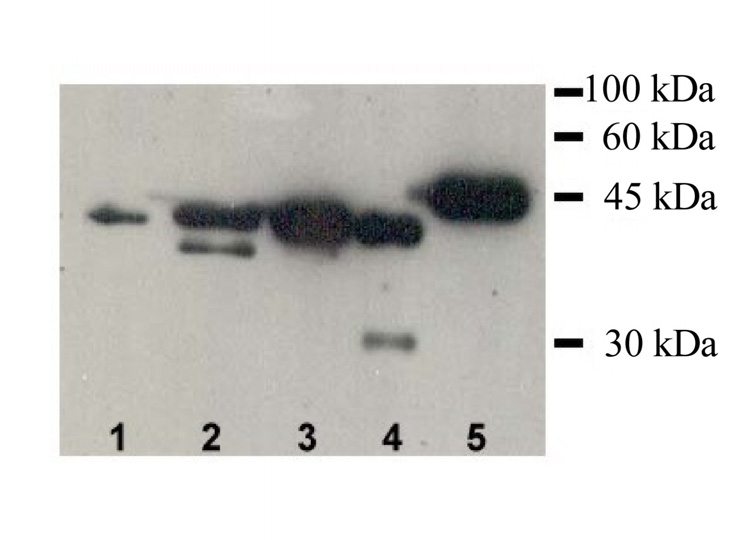
Western blot analysis performed using an anti β-DG antibody versus a DGC enriched protein extract of skeletal muscle from different teleost species (4 μg for each lane). Lane: 1) *D. labrax*; lane 2) *T. nigroviridis*, lane 3) *T. bernacchii*; lane 4) *C. hamatus*; lane 5) *D. rerio*.

To evaluate the expression of DG in the species analysed, skeletal muscle total protein extracts, partially purified upon a WGL-enrichment protocol (as described in Methods), were tested via Western blot, and the presence of β-DG was revealed using the commercially available monoclonal antibody anti-β-DG 43DAG. This antibody is able to recognize the last portion (15 aa) of the C-terminal cytodomain of β-DG, harbouring the dystrophin binding site [17] (see Fig. 1). The identified band of ≈ 43 kDa clearly corresponds to β-DG and confirms its expression in all the samples analysed (Fig. 3). A small mobility shift was recorded that could depend on slight differences in the levels of glycosylation of the β subunit. In *T. nigroviridis *two bands for β-DG were detected while an additional ≈ 30 kDa band was identified in *C. hamatus *(Fig. 3). It is noteworthy that in mammals a proteolytic fragment of the same size was related to a series of severe pathologies, including cancer progression [18,19]. At the present stage, we could not assess whether such fragment would originate from some proteolytic events or whether it would be an alternative expression product of a putative paralogous copy of *DAG1 *in this species.

## Discussion

During recent years, the biological role of dystroglycan (DG) in higher vertebrates has been in part elucidated. The DG adhesion complex, composed of two subunits (α and β), is a pivotal member of a large transmembraneous group of glycoproteins associated with the cytoskeleton representing, together with integrins, the major molecular bridge involved in the formation and stabilization of contacts at the cell/extracellular matrix interface during embryogenesis and in a wide variety of adult tissues [8,17]. In mice, the concerted action of DG and laminin is believed to trigger the initial phase of embryogenesis, when the first contacts between cells and basement membranes are established. In fact, *DAG1 *knockout mice exhibit gross developmental abnormalities beginning around 6.5 days of gestation, while in contrast heterozygous mice are viable and fertile [8]. However, the role of DG during embryogenesis remains controversial. Although no mutations have been identified so far in human populations, thus confirming the DG crucial primary role during peri-implantation in mammals, knock-out experiments in zebrafish showed that early development remained unaffected by the absence of DG while a severe dystrophic phenotype emerged during adulthood [5].

The comparison of *DAG1 *among different vertebrate species, including several fish species and even antarctic ones, which typically underwent the evolutionary process of cold-adaptation, could be useful to understand how the selection pressure influenced the actual organization of *DAG1 *in fish and the whole genome duplication process. In fact, several lines of evidence suggest that a whole-genome duplication (WGD) event occurred within the teleost lineage after separation from the tetrapod lineage, and that only a subset of duplicates have been retained in modern teleost genomes [16]. The analysis of genomic sequences obtained from zebrafish and pufferfish provided further evidence for WGD during the evolution of ray-finned fish (Actinopterygii) [16,20]. It was estimated that WGD should have taken place about 350 Myr ago, after the separation of ray-finned and lobe-finned fish, but before the origination of teleost fish [21]. While several duplicated genes were subsequently lost, many others were maintained during evolution. Preserved genes might have underwent small changes and adopted slightly different functions and this might have further protected the gene from being lost [22,23]. These assumptions are of primary importance when searching for possible orthologous versions of mammalian genes in fish genomes [24,25].

The major piece of data collected so far on the structure and function of *DAG1 *in zebrafish is the work published by Parsons and colleagues, which shows that the inactivation of the DG gene by antisense morpholino oligonucleotides causes severe muscular dystrophy in the adult stage [5]. Genome analysis reveals that only one copy of *DAG1 *is present in *D. rerio*, displaying the typical uncomplicated exon/intron mammalian structure [26]. On the other hand, the analysis of available genomic sequence drafts from *T. rubripes*, *T. nigroviridis*, *O. latipes *and *G. aculeatus*, reveals the presence of two ORFs encoding DG, that we here name as *DAG1a *and *DAG1b*, based on their alignment scores with respect to other mammalian DGs and in particular to human DG (see Fig. 1, Table 1 and 2).

Surprisingly, the gene copy that we propose to define *DAG1a*, displays a novel intronic sequence at the level of the region corresponding to the second exon. The intron is very short in size: 137 bp in *T. rubripes and T. nigroviridis*, 116 bp in *G. aculeatus and D. labrax *and only 86 bp in *O. latipes *(see Table 1) in close similarity with the shortest sizes of introns already identified in other species [27]. The gain of this "mini-intron" did not produce any frameshift affecting the resulting protein sequence, as also demonstrated by our Western blot results (see below). Accordingly, experiments performed with specific primer pairs designed for both *DAG1a *and *DAG1b*, reveals that in pufferfish both the *DAG1 *copies are transcribed and therefore likely to be functional and expressed (Fig. 2). This result was somehow anticipated by the high conservation of both paralogous *DAG1 *sequences and by the absence of nonsense mutations or any other major genetic alteration that would imply a drift towards a pseudogene status. In fact, pseudogenes are known to constantly drift until they are either deleted or become unrecognizable [28]. However, further analysis will be needed to investigate in detail such intron gaining event [29].

**Figure 4:**
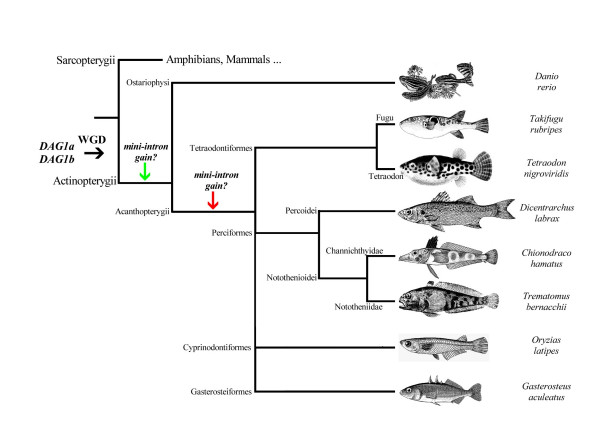
Phylogenetical tree of the different fish species under analysis built using the taxonomical information available at the NCBI database. Origin of fish pictures: the images have been downloaded from freely available web resources and extensively readapted. The black arrow represent the whole genome duplication (WGD) event, involving also the DG gene, that took place during the Actinopterygii speciation. The two additional arrows (green and red) mark hypothetical timings of the mini-intron gaining event.

As already reported for several other genes, it is likely that *DAG1 *underwent duplication as part of the whole genome duplication (WGD) event that took place during the Actinopterygii speciation process [16,25] (black arrow in Fig. 4) and subsequently a sporadic gain of a mini-intronic sequence took place either before the separation between Ostariophysi and Acanthopterygii (green arrow in Fig. 4) or afterwards (red arrow).

In *D. labrax *(sea bass), the result of our homologous cloning strategy for DG fishing was a gene fragment of ≈ 2000 bp (data not shown), including a sequence corresponding to a 116 bp mini-intron which, based on the alignment score, can be assigned to the family of *DAG1a *sequences (Table 3). The expression of DG was preliminary tested by Western blot using a monoclonal antibody directed versus the C-terminal tail of the β-DG subunit, since this region is highly conserved in all the vertebrates [30]. In fact, positive signals of 43 kDa were detected in all the samples analysed, including antarctic species [13-15]. Up to now, any attempt at homologous cloning of DG sequences from antarctic species exploiting the same primers employed for *D. labrax *DG were unsuccessful. Therefore, further experiments employing new designed degenerate primers will be required in order to clone the DG sequences from antarctic species.

The secondary structure of the α-DG N-terminal region of *T. rubripes*, predicted from the gene sequence (both *DAG1a *and *DAG1b*) exploiting SSpro software [31,32] (data not shown), suggests a significant similarity with the α-helical and β-strand elements detected in the crystal structure of mouse α-DG N-terminal domain that was recently solved [33]. This region is composed by two autonomous domains: an Ig-like one, and the second one resembling ribosomal RNA-binding protein S6. Moreover, additional predictions performed using NetOGlyc software, confirm the presence of a mucin-like domain also in the central region of teleost α-DGs. It is noteworthy that the β-DG binding epitope, spanning the amino acid positions 550–565 of the C-terminal domain of α-DG, is highly conserved also in fish [34]. In contrast, its counterpart, the putative α-DG binding epitope spanning the amino acidic positions 691–719 in β-DG, displays a much lower degree of identity with the mammalian one, with few exceptions, such as the conservation of Phe692 and Phe718, which have been shown to play a crucial role in the α/β subunits interface formation (Fig. 1) [35]. Another region highly conserved is the C-terminal domain of β-DG which contains the dystrophin binding site [17,30,36].

## Conclusion

Our analysis clearly shows that the WGD event that took place in Actinopterygii involved also *DAG1*. During evolution, WGD events are expected to have had a high impact on speciation. To fully understand this impact means to unravel all the genetic and molecular details underlying the speciation process, and the knowledge of which genes were retained in duplicate and how the duplication modified their evolutionary fitness is crucial to that aim. Generally, the functional consequences of WGD in fish have been mitigated both by partial gene loss and acquisition of new useful functions [37]. Indeed, in some cases the presence of two functional copies of an important gene like DG, could have represented an improvement of their fitness. The morpholino oligos-driven disruption of DG in zebrafish results in the emergence of a severe dystrophic phenotype in adults that could not be alleviated or compensated by a paralogue isoform of DG [5].

The importance of fish model systems for the study of Duchenne muscular dystrophy and other human muscular diseases is clearly emerging, as highlighted by the work carried out in Kunkel's lab [38]. Due to the important role played by DG in human congenital muscular disorders, comparative genetic and biochemical analyses could be particularly relevant in the race for fully elucidating its function or misfunction in severe diseases, eventually leading to innovative therapeutical strategies related to DG. For example, the DG's high affinity towards the proteoglycan agrin has been one of the factors leading to the design of miniaturized agrin, rescuing the dy/dy dystrophic phenotype in mice [39,40].

A comprehensive understanding of the biological implications of *DAG1 *duplication in some teleost fish species may have unexpected repercussions on the view of "secondary dystroglycanopathies", since recently paralogue isoforms of glycosyltransferases thought to act specifically on DG (LARGE and POMT among others) were also identified and characterized [41,42]. It is intriguing to hypothesize that in the future "evolution-inspired" gene therapy approaches, implying the introduction (or reintroduction) of a second *DAG1 *copy or isoform, will be used to alleviate the symptoms of dystrophy in human skeletal muscle.

## Methods

### Nucleic acids extraction and cDNA production

Genomic DNA was obtained by Sodium acetate/Chloroform extraction from tissues previously homogenised in a solubilization buffer and digested with Proteinase K (Sigma-Aldrich, USA). The Abs_260 _and Abs_260_/Abs_280 _ratios were used to determine DNA concentration and purity, respectively.

Total RNA was extracted from tissues using the RNeasy Mini kit (Qiagen, Germany) specific for fibrous tissue. Using this protocol we observed a massive contamination with genomic DNA co-eluting with RNA. To avoid such contamination, a further step was added by loading the aqueous phase on an RNeasy column (Qiagen, Germany) and before elution the membrane was treated with DNase I. First strand cDNA was then synthesized using the Enhanced Avian RT-PCR kit (Sigma-Aldrich, USA) and used as a template for further PCR experiments.

### PCR analysis and gene cloning

All the PCR reactions (50 μl volume) were performed in a GeneAmp PCR System 2400 temperature cycler (Perkin Elmer) using 10 ng of cDNA as template, and 2.5 U of AccuTaq DNA polymerase (Sigma). As positive control we used, for each species, specific primers that amplify the actin gene (Table 3). PCR products were cloned into the pCR II-TOPO vector (Invitrogen) using a TOPO TA Cloning^® ^kit following the manufacturer's protocol. Plasmidic DNAs containing the cloned inserts were purified and submitted to automated sequencing. All the primers used in this analysis are reported in Table 3. The primers FISH_ext_s and FISH_ext_as were designed on two highly conserved regions identified aligning the sequences of *DAG1 *from *D. rerio *and *T. rubripes*. For *T. nigroviridis *we designed two primer pairs specific for *DAG1a *and *DAG1b*, respectively (Table 3). The specific primers ACT_s and ACT_as for actin genes of all the species where designed with an optimum GC concentration and an annealing temperature of 55°C. In order to distinguish the amplified product from genomic DNA and from cDNA, ACT primers were designed into contiguous exons. The newly identified DG sequence of *D. labrax *has been deposited in Gene Bank and assigned the accession number DQ149510.

### Sequence analysis

The multiple alignment of all the DG protein sequences under analysis has been obtained using ClustalW [43]. Based on their respective primary structure, a secondary structure prediction of the N-terminal region of the *DAG1a *and *DAG1b *products from *T. rubripes *was obtained using the program SSpro [31,32]. The propensity of an amino acidic stretch to be O-glycosylated was analysed using the NetOGlyc software [44].

### Total protein extracts and WGL-enriched fraction preparation from tissues

Total protein extracts were obtained from skeletal muscle biopsies in the form of freshly frozen samples collected from all the species under analysis and stored at -80°C until used. Tissue samples were homogenized in a solubilization buffer (50 mM Tris-HCl pH 7.4, 1 mM EDTA, 1 mM DTT, 1% SDS) and centrifuged to obtain a clean upper phase that was successively incubated with Wheat germ lectin (WGL) Sephorose 6 MB (Amersham, Sweden) to obtain DGC [45]. Western blot analysis of tissue samples were performed as described elsewhere [45].

## Authors' contributions

EP did all the experimental work including the collection, storage and homogenization of the fish tissue samples and also gave a major contribution to the writing of the manuscript and assembly of the final version of figures and tables. DC carried out some of the PCR experiments dealing with the identification of two expressed sequence in cDNA and readapted the fish pictures. MO carried out extensive sequence alignment analysis. RT carried out the Western blot analysis. AG and BG contributed to edit the manuscript. AB conceived the project, supervised the experiments and wrote the manuscript together with EP. All authors read and approved the final manuscript.

## Figures and Tables

**Table 1:** DG genes details based on genomic DNA sequences available at the Ensembl databank

				**gene structure exon/intron (bp)**						
**SPECIES**	**GENE**	**ID ENSEMBL**	**GENOME LOCATION**	**exon1**	**intron1**	**exon2**	**mini-intron**	**exon3**	**total length (bp)**	**precursor (aa)**

*H. sapiens*	*DAG1*	ENSG00000173402	chromosome 3	285	19977	2403	no	no	22665	895
*M. musculus*	*DAG1*	ENSMUSG00000039952	chromosome 9	279	8351	2403	no	no	11033	893
*D. rerio*	*DAG1*	ENSDARG00000016153	chromosome 22	321	15186	2280	no	no	17787	866
*T. rubripes*	*DAG1a*	NEWSINFRUG00000153220	scaffold 2682	336	2938	435	137	> 1689	> 5674	> 820
*T. rubripes*	*DAG1b*	NEWSSINFRUG00000136139	scaffold 75	366	818	2307	no	no	3491	890
*T. nigroviridis*	*DAG1a*	GSTEN00009405001	scaffold SCAF11228	?	> 606	441	137	1857	> 3041	> 765
*T. nigroviridis*	*DAG1b*	GSTENG00033210001	scaffold SCAF15033	369	779	2313	no	no	3461	893
*O. latipes*	*DAG1a*	ENSORLG00000010935	chromosome 5	297	22628	459	86	1818	25288	857
*O. latipes*	*DAG1b*	ENSORLG00000009878	chromosome 7	312	839	2283	no	no	3434	864
*G. aculeatus*	*DAG1a*	ENSGACG00000003062	group XVII 379.49 k	?	?	462	116	1827	> 2405	> 762
*G. aculeatus*	*DAG1b*	ENSGACG00000002661	group XII 444.80 k	312	1026	2301	no	no	3639	870
*D. labrax*	*DAG1a*	NCBI accession code DQ149510	?	?	?	> 90	116	> 1783	> 1989	> 624

Details relative to the newly identified partial DG sequence from *D. labrax *are also included.

**Table 2:** Reciprocal alignment scores of human and fish *DAG1 *genes obtained with ClustalW

	***H. sapiens DAG1***	***D. labrax DAG1a***	***T. nigroviridis DAG1a***	***T. rubripes DAG1a***	***O. latipes DAG1a***	***G. aculeatus DAG1a***	***T. nigroviridis DAG1b***	***T. rubripes DAG1b***	***O. latipes DAG1b***	***G. aculeatus DAG1b***
*H. sapiens DAG1*	-	66	64	55	57	64	53	53	54	55
*D. labrax DAG1a*	66	-	**84**	**80**	**80**	**90**	68	68	64	69
*T. nigroviridis DAG1a*	64	**84**	-	**77**	**73**	**79**	63	63	60	66
*T. rubripes DAG1a*	55	**80**	**77**	-	**68**	**74**	58	57	55	57
*O. latipes DAG1a*	57	**80**	**73**	**68**	-	**78**	57	57	55	58
*G. aculeatus DAG1a*	64	**90**	**79**	**74**	**78**	-	64	63	62	65
*T. nigroviridis DAG1b*	53	68	63	58	57	64	**-**	**90**	**75**	**80**
*T. rubripes DAG1b*	53	68	63	57	57	63	**90**	**-**	**74**	**79**
*O. latipes DAG1b*	54	64	60	55	55	62	**75**	**74**	**-**	**75**
*G. aculeatus DAG1b*	55	69	66	57	58	65	**80**	**79**	**75**	**-**

The *DAG1a *and *DAG1b *orthologues have been highlighted in bold.

**Table 3:** Primers used in the PCR experiments

**Fish**	**Primer**	**Sequence primer (5'-3')**
*D. rerio*	DAG1_s	CCAGCCTTTCATCTGTGGCAA
*D. rerio*	DAG1_as	CTTCGCACCCTTTTGGGCAC
*D. rerio*	ACT_s	TCTTGACCCTGAAGTACCCCATT
*D. rerio*	ACT_as	TCCTTGATGTCGCGCACAAT
*D. labrax*	FISH_ext_s	GGGCTTCAGCACATGAAGAT
*D. labrax*	FISH_ext_as	CTGTAGGG(A/G)GTCATGTTCTT
*D. labrax*	ACT s	TCCTGACCCTGAAGTACCCCA
*D. labrax*	ACT as	TTGATGTCACGCACGATTTCC
*T. nigroviridis*	DAG1a s	CAGACGTTCCTGTGTGAGGGG
*T. nigroviridis*	DAG1a as	GCTTCGGAAGGTGCTGCTTC
*T. nigroviridis*	DAG1b s	AGCTCAGCCTCTCACCTGTAGC
*T. nigroviridis*	DAG1b as	GACTCGTTTCACTCCATGGACC
*T. nigroviridis*	ACT s	CACCCTGAAGTATCCCATCGAA
*T. nigroviridis*	ACT as	GTCTCTGACGATCTCTCGCTCAG

The primers used to amplify the gene sequences from *D. rerio *and *T. nigroviridis *were designed using the sequences available in the database. The degenerate primers FISH_ext_s and FISH_ext_as, which allowed the fishing of the newly identified *D. labrax *sequence, were chosen exploiting two regions displaying very high homology within the aligned DG sequences from *D. rerio *and *T. rubripes *(see also Fig. 1).
